# Phenotyping and characterising gait profiles of people with multiple sclerosis

**DOI:** 10.1038/s41598-025-19559-6

**Published:** 2025-09-17

**Authors:** Katrin Trentzsch, Dirk Schriefer, Heidi Stölzer-Hutsch, Hernan Inojosa, Tjalf Ziemssen

**Affiliations:** Center of Clinical Neuroscience - Neurological Clinic, Faculty of Medicine and University Hospital Carl Gustav Carus, TUD Dresden University of Technology, 01307 Dresden, Germany

**Keywords:** Phenotyping, Gait, Pattern detection, Gait or walking classification, Multiple sclerosis, Neuroscience, Medical research, Neurology, Signs and symptoms

## Abstract

Gait disorders are among the most disabling symptoms in people with multiple sclerosis (pwMS), substantially affecting quality of life, autonomy, and participation. The Expanded Disability Status Scale (EDSS) captures impairments -particularly pyramidal, cerebellar and sensory- that influence gait patterns. Quantitative gait analysis enables objective exploration of these patterns supporting personalised strategies. This study aimed to characterise gait profiles in pwMS based on EDSS-derived functional scores. In this cross-sectional study at University Hospital Dresden, 204 pwMS and 237 healthy controls underwent instrumented gait assessments under self-selected, fast, and dual-task conditions. PwMS were categorised into pyramidal, cerebellar, or sensory gait pattern groups based on EDSS functional system scores. Spatiotemporal gait parameters were analysed using generalised linear models adjusted for age, gender, and body mass index. Sensory gait patterns were observed in 57.8% of pwMS, pyramidal in 27.9%, and cerebellar in 14.2%. Significant group differences emerged across all walking conditions. Ataxic gait showed reduced speed and increased step and stride variability. Sensory patterns featured higher speed, longer steps, and increased cadence. Pyramidal patterns resembled ataxic ones but differed under fast walking. EDSS-based functional deficits correspond to distinct MS-specific gait profiles. Multidimensional clinical gait phenotyping provides actionable insights for tailoring rehabilitation and monitoring mobility outcomes in pwMS.

**Supplementary Information:**

The online version contains supplementary material available at 10.1038/s41598-025-19559-6.

## Introduction

Multiple sclerosis (MS) is an autoimmune demyelinating disease of the central nervous system that leads to progressive disability due to axonal loss and dysfunction of the several neurological systems^1,2^. 70% of people with MS (pwMS) who experience difficulty walking reported this as the most challenging aspect of their condition^3^. Gait abnormalities in pwMS exhibit considerable heterogeneity, driven primarily by spasticity, sensory disturbances, ataxia, balance impairments, and fatigue^4–6^. As a result, gait impairment in MS is both multifactorial and highly individualised, requiring detailed and multidimensional assessment approaches.

The Expanded Disability Status Scale (EDSS) is a well-established clinical tool and the standard instrument used in clinical trials to assess disease progression and neurological dysfunction in MS. It enables detailed evaluation of specific clinical impairments through its functional systems, such as pyramidal, cerebellar, and sensory systems. These impairments are reflected in characteristic gait patterns: pyramidal disorders can lead to spastic gait patterns with limited range of motion and asymmetry; cerebellar deficits are often associated with gait ataxia and incoordination; sensory deficits may result in increased instability^7^. Analyzing these EDSS-associated gait impairments offers valuable clinical insights and forms the basis for symptom-specific therapeutic strategies. However, this clinical classification remains limited by the inherent subjectivity of the EDSS, its dependence on clinician rating, and its insufficient sensitivity to subtle or multidimensional gait abnormalities^8^. Implementing standardized digital gait analysis could help address these shortcomings by providing more objective, consistent, and comprehensive measurements of gait patterns.

Previous studies have explored the relationship between MS-related disability and gait characteristics, often using general EDSS scores or MS disease subtypes (e.g. relapsing vs. progressive) as group-defining variables^9–14^. However, the classification of functional deficits was often limited, and the complexity of spatiotemporal gait parameters across different walking conditions was not always fully addressed. Kalron et al., for instance, identified differences in gait related to functional system involvement but relied on broad EDSS categories^7^. Filli et al. later applied cluster analysis to gait kinematics and identified three main pathological gait types in pwMS, but their classification was not anchored in clinical functional system scores^15^. These studies underscore important associations between MS pathology and gait abnormalities, but often lacked integration of detailed neurological assessments and patient-reported outcomes, as well as comparisons with healthy controls. Moreover, many did not assess spatiotemporal gait parameters under challenging walking conditions such as fast walking or dual-tasking, which may reveal subtle motor and cognitive deficits. Cognitive function plays a critical role in gait control, particularly under complex conditions. Evidence from MS and other neurological disorders suggests that impairments in executive function and attentional resources are associated with increased gait variability and instability^16,17^.

The aim of this study is to classify gait patterns in pwMS based on detailed EDSS-derived functional system scores, specifically addressing impairments in the pyramidal, cerebellar, and sensory systems. We examine how these clinically defined groups differ in terms of demographic and disease-related factors, patient-reported mobility outcomes, and spatiotemporal gait characteristics. Gait was assessed under three walking conditions - self-selected speed, fast walking, and dual-task - to reflect different physical and cognitive demands that pwMS may encounter in daily life. Standardised visualisation tools, such as radar plots, support the identification of clinically relevant gait profiles. By linking neurological examination findings to quantitative gait data and patient-reported experiences, our study aims to enhance the precision of MS gait phenotyping and support the development of pattern-specific rehabilitation strategies.

## Methods

### Study design

**Fig. 1:**
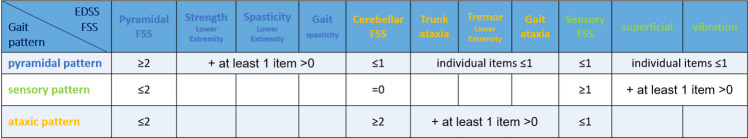
Classification criteria for gait patterns based on the functional systems of the EDSS. This figure presents the multivariate classification criteria the three MS gait pattern groups: pyramidal gait pattern, ataxic gait pattern, and sensory gait pattern, defined by the EDSS functional systems Pyramidal, Cerebellar, and Sensory. Pyramidal impairments were assessed using the subscores for lower limb strength and spasticity and spasticity during walking. Cerebellar impairment was assessed by the subscores for trunk ataxia, tremor of the lower extremities and gait ataxia. Sensory impairment was defined by limitations in surface sensitivity or vibration perception. Only patients who met both the main functional system and subscore criteria were included in this study. The group definitions are mutually exclusive, meaning each patient could be assigned to only one gait pattern group. (EDSS = Expanded Disability Status Scale; FSS = Functional System Score; pwMS = People with Multiple Sclerosis).

We performed a cross-sectional study at the MS Centre of the Centre for Clinical Neuroscience of the Department of Neurology of the University Hospital Carl Gustav Carus, Dresden, Germany. PwMS and healthy controls (HC) without neurological disease were invited to participate, with recruitment conducted between May 2018 and March 2023. The inclusion criteria for pwMS comprised: (1) a confirmed diagnosis of MS according to the 2017 revisions of the McDonald criteria^18^, (2) the ability to walk without aid, (3) an EDSS score of ≤ 5.5 (able to walk 100 m without a rest) and a fulfilment of the specific EDSS inclusion criteria based on assessments of the pyramidal, cerebellar and sensory functional systems as detailed in Fig. 1, and (4) provision of signed informed consent. All patients were relapse-free for at least 3 months prior to study inclusion. PwMS with medical conditions that could negatively affect gait (e.g. orthopaedic diseases, pregnancy) at the time of examination were excluded. All study participants underwent gait analysis following the Dresden Protocol for Multidimensional Walking Assessment (DMWA)^19^. This study was reviewed and approved by the Ethics Committee at Technische Universität Dresden (approval number BO-EK-320062021).

### Inclusion criteria according to Expanded Disability Status Scale (EDSS)

All pwMS were clinically examined by an EDSS Neurostatus-certified examiner, and the EDSS Neurostatus was used to quantify the degree of disability. The EDSS, a reference measure of neurological disability in MS, evaluates eight functional systems (FS): visual, brainstem, pyramidal, cerebellar, sensory, bowel and bladder, cognition/fatigue, and walking ability. Each functional system is scored on a scale from 0 (no disability) to 5 or 6 (maximum disability), and these contribute to an overall score ranging from 0 (neurologically normal) to 10 (death due to MS). A score of 0 indicates “neurologically normal”, a score of 1 indicates minimal, subclinical abnormalities, and a score of 2 reflects at least a clinically relevant disability^20^.

All EDSS examinations were conducted by three Neurostatus-certified raters. To minimize variability, only three raters were involved throughout the study, and in cases of uncertainty, classification was resolved by consensus or the patient was excluded from group assignment.

PwMS were classified into one of three gait pattern groups (pyramidal, sensory, ataxic) based on the scores and subscores of the pyramidal, cerebellar, and sensory function systems. Figure 1 provides a detailed overview of the classification criteria. To ensure that only impairments affecting the lower extremities were considered, specific subscores of FS were included in the classification process. For the pyramidal FS, we evaluated the EDSS subscores for strength, spasticity of the lower extremities, and gait spasticity. Similarly, for the cerebellar FS, we incorporated the EDSS subscores for trunk ataxia, lower extremity tremor, and gait ataxia. For the sensory functional system, classification required the presence of limitations in sensitivity to superficial or vibration stimuli. In our experimental approach, we selected only those patients who strictly met the inclusion criteria for the three phenotypes.

This classification approach was consistently applied by the same three raters across all participants, with consensus procedures used to resolve borderline cases, ensuring reproducibility and minimizing interrater variability.

### Gait analysis

Multidimensional gait analysis involves the systematic recording of human gait at different speeds under differentiated tasks using standardised test procedures^19^. The spatiotemporal gait parameters were recorded using the GAITRite system (CIR-Systems, Franklin, NJ, United States). The instrumented walkway is equipped with pressure sensors that record pressure during walking. It measures 884 cm × 90 cm × 0.32 cm^21^.

All participants were instructed to perform the following gait protocol:

- Walking at a self-selected speed in one direction across the mat without stopping (2 trails).
- Walking with the fastest possible speed in one direction across the mat without stopping (1 trail).
- Walking under dual task conditions (with standardised verbal fluency and calculation tasks) (2 trails).

For normal walking, an average of 22.86 ± 7.75 steps was analysed per trial, while 24.82 ± 8.44 steps were considered during dual-task walking and 9.38 ± 2.51 steps during fast walking (values represent mean ± SD). To ensure robust gait pattern evaluation, all valid steps were included in the analysis, while invalid steps were excluded.

Self-selected and dual-task walking were performed twice, while fast walking was conducted only once. The dual-task condition was repeated to account for higher intra-individual variability, ensuring a reliable assessment of gait adaptation under cognitive load. Self-selected speed was also measured twice to capture natural variability in preferred walking pace. In contrast, fast walking was only assessed once, as it provides a maximal-effort measure that typically shows lower intra-individual variation. The respective mean values of the individual gait variables were used for the analyses.

Gait analyses under dual task conditions may detect mobility restrictions in gait instability at an early stage by allowing participants to perform cognitive tasks simultaneously^22–24^. Gait testing under dual task conditions were carried out with two tasks (a verbal fluency task and a calculation task).

Quantitative gait assessment evaluates pwMS performance across several gait parameters. These were classified into gait domains based on established models (Table 1)^25–28^.

**Table 1:** Gait domains with underlying gait variables.

Associated gait domain	Gait variable	Unit	Interpretation
Pace	Velocity	cm/s	higher = “better”
	Step length	cm	higher = “better”
	Stride length	cm	higher = “better”
	Double support time	s	lower = “better”
Rhythm	Cadence	step/min	higher = “better”
	Step time	s	lower = “better”
	Swing time	s	lower = “better”
	Stance time	s	lower = “better”
Variability (SD)	Stride length variability	cm	lower = “better”
	Swing time variability	s	lower = “better”
	Stance time variability	s	lower = “better”
	Step length variability	cm	lower = “better”
Asymmetry (differential)	Step time asymmetry	s	lower = “better”
	Cycle time asymmetry	s	lower = “better”
	Step length asymmetry	s	lower = “better”
	Base of support	cm	shorter = “better”

This table summarizes the 16 key gait variables chosen for this study, informed by existing literature on gait domains. It outlines their units and interpretation guidelines including classification into overarching gait domains. For example, the rhythm domain captures variables that describe the regularity of gait, while the pace domain focuses on variables related to the speed and cadence of walking. The direction of interpretation highlights whether improved performance is indicated by higher or lower values. All variability variables were calculated using the standard deviation and asymmetry variables using the absolute difference between left/right difference values of the variable (exception: base of support); SD = Standard Deviation.

This was followed by three additional clinical function tests, including the Timed 25-Foot Walk Test (25FWT), the 2-Minute Walk Test (2 MWT) and the 3 m Backward Walking Test (3MBWT). The 25FWT, one of the most widely used and robust clinical test for evaluating walking speed, measured the time required to walk 7.62 m, recorded by a nurse using a stopwatch^29^. Walking endurance was evaluated using the 2MWT, where the total walking distance over 2 min was continuously recorded with an odometer^30,31^. The 3MBWT, a validated and reliable method for assessing backward walking ability in pwMS, serves as a predictor of fall risk and complements the assessment of walking impairments^32,33^.

In addition to the sensor-based gait analysis, the patient’s perception of walking impairment was assessed using two patient-reported outcomes (PROs) measures, the 12-item Multiple Sclerosis Walking Scale (MSWS-12) and the 9-item Early Mobility Impairment Questionnaire (EMIQ)^34,35^. These PROs provide valuable insights into patients’ perception of walking limitations, complementing clinical gait data and contributing to a more comprehensive understanding of the impact of gait impairment on daily life.

### Statistical methods

The demographic and disease-related characteristics of the study participants, along with the outcomes of the gait analysis, were summarised using means, standard deviations, medians, frequencies, and percentages. Differences between the gait pattern groups in terms of demographics and disease-related characteristics were analysed using (Bonferroni-adjusted) chi-square tests for categorical variables, and Kruskal-Wallis and Bonferroni-adjusted Mann-Whitney U-tests for continuous and ordinal variables. The distribution of gait outcome parameters was evaluated visually using histograms and quantile-quantile plots, and supplemented by the Shapiro-Wilk test. The association between spatiotemporal gait outcomes (dependent variables) and the gait pattern groups (ataxic, sensory, and pyramidal) was assessed using multivariable generalized linear models (GLM), adjusted for age, sex, and body mass index. The variables gait pattern group, age, sex, and body-mass-index were designated as fixed main effects. Disease duration was not included as an additional covariate due to its strong correlation with age and EDSS-based disease severity (used for group classification). Furthermore, no significant group differences in disease duration were observed (Table 2). Data inspection determined the appropriate distribution and link function of the GLM as follows: Normally distributed variables were analysed using a Gaussian distribution with an identity link function. Right-skewed data were analysed using a gamma distribution and log link function. In the event of a statistically significant main effect for pattern group, post-hoc pairwise comparisons (contrast tests) were conducted with Bonferroni corrections to adjust for multiple comparisons. Analyses of the spatiotemporal gait variables were conducted on a condition-by-condition basis (self-selected walking speed, fast walking, and dual-task walking). Participants who did not complete one of the three conditions were not excluded from the study. Instead, the sample size (N) for that specific condition was reduced, and analyses were conducted using the available data for each condition. No imputation was performed for missing data. Statistical significance was defined as *p* < 0.05. All calculations were performed using IBM SPSS version 29.0 (IBM Corporation, Armonk, NY, USA). Bar chart figures were generated with GraphPad Prism version 10 (GraphPad Software Inc., La Jolla, CA, USA), while radar plots were created with Microsoft Excel version 2016 (Microsoft Corporation, Redmond, WA, USA).

### Radar plot

Radar plots were used to visualise the ataxic, sensory, and pyramidal gait patterns of pwMS by displaying the spatiotemporal gait metrics in a spider web format. For this purpose, all 16 gait parameters for pwMS were standardised relative to the control group using z-scores, resulting in a dimensionless, comparable measure. The z-score for each spatiotemporal parameter was calculated using the following formula:

z = (X - mean of controls) / standard deviation of controls.

where X represents the observed value for each pwMS. The radar plots illustrate the mean z-scores for each group, with the magnitude of the z-score indicating the degree of deviation (in standard deviation units) from the control group. A positive z-score indicates a value above the control group mean, while a negative z-score reflects a decrease in value (see Table 1 for directionality of parameter interpretation).

Radar plots have been shown to be a promising tool for visualising multidimensional gait data and aiding in the diagnosis and monitoring of gait impairment in various populations and conditions^36^.

## Results

### Participants

A total of 204 pwMS (age: 42.11 ± 11.18; BMI: 25.07 ± 4.45; disease duration: 10.31 ± 7.35; EDSS median: 2.0) met the inclusion criteria and were assigned to one of three distinct gait pattern groups. From these, 57.8% of the pwMS (*n* = 118) showed a sensory gait pattern. A pyramidal pattern was assigned to 27.9% (*n* = 57) and an ataxic pattern to 14.2% (*n* = 29) of the pwMS. Participant characteristics, including disease duration and ongoing therapy status for each group, are presented in Table 2. The HC group (*n* = 237) included 63.3% (*n* = 150) women with a mean age of 34.64 ± 10.97 years and a BMI of 23.86 ± 4.19.

**Table 2:** Characterization of patient demographics and clinical features by gait pattern subgroups (N = 204).

	Pyramidal pattern	Ataxic pattern	Sensory pattern	*p*-value
N	57	29	118	
Females (N; %)	50 (87.7%)	21 (72.4%)	86 (72.9%)	0.076
Age (years)	45.93 ± 11.63	44.62 ± 12.14	39.64 ± 10.10	0.001^b^
Body mass index (kg/m²)	24.61 ± 4.18	24.68 ± 3.35	25.39 ± 4.81	0.657
Disease duration (years)	11.50 ± 7.48	12.28 ± 9.26	9.21 ± 6.55	0.109
EDSS (median, IQR)	2.5 (IQR 2.0-3.5)	3 (IQR 2.25–3.5)	1.5 (IQR 1.5-2.0)	< 0.001^a, b^
Pyramidal	2 (IQR 2–3)	2 (IQR 1–2)	1 (IQR 1–1)	<0.001^a, b,c^
Sensory	1 (IQR 0–1)	0 (IQR 0–1)	1 (IQR 1–2)	< 0.001^a, b^
Cerebellar	1 (IQR 1–1)	2 (IQR 2–2)	0 (IQR 0–0)	<0.001^a, b,c^
MS type (N; %)				
RRMS	48 (84.2)	23 (79.3)	116 (98.3)	
SPMS	2 (3.5)	2 (6.9)	0	
PPMS	7 (12.3)	4 (13.8)	2 (1.7)	
DMT (N; %)				
Basic DMTs	14 (24.6%)	3 (10.3%)	44 (37.3%)	
Moderately effective DMTs	8 (14.0%)	3 (10.3%)	14 (11.9%)	
Highly effective DMTs	21 (36.8%)	7 (24.1%)	35 (29.7%)	
No DMT	12 (21.1%)	14 (48.3%)	19 (16.1%)	
Unknown	2 (3.5%)	2 (6.9%)	6 (5.1%)	

Summary of patient demographics and disease-related clinical information, categorized according to the three distinct gait pattern subgroups based on the FSS, as illustrated in Fig. 1. The results are given as mean ± SD unless otherwise stated. Distribution of disease-modifying therapies (DMTs) by gait phenotype, categorized according to efficacy levels. Basic DMTs include interferons, glatiramer acetate, teriflunomide, and fumarates. Moderately effective DMTs include fingolimod, ozanimod, and cladribine. Highly effective DMTs include monoclonal antibodies such as natalizumab, ocrelizumab, and alemtuzumab. No DMT: patients without active treatment and Unknown is unclassified. The classification reflects efficacy levels commonly used and aligned with the categories of the German MS guideline^37^. Global comparisons among the three gait pattern groups were conducted using the Kruskal-Wallis test for continuous variables and the Chi-squared test for categorical variables. Statistically significant differences in post-hoc tests between gait pattern groups (pairwise comparisons) are indicated by superscripts in the p-value column: ^a^sensory-ataxic, ^b^sensory-pyramidal, and ^c^ataxic-pyramidal (Bonferroni adjusted Mann-Whitney U test or chi-square test); EDSS = Expanded Disability Status Scale; RRMS = relapsing remitting MS; SPMS = secondary progredient MS; PPMS = primary progressive MS.

**Fig. 2:**
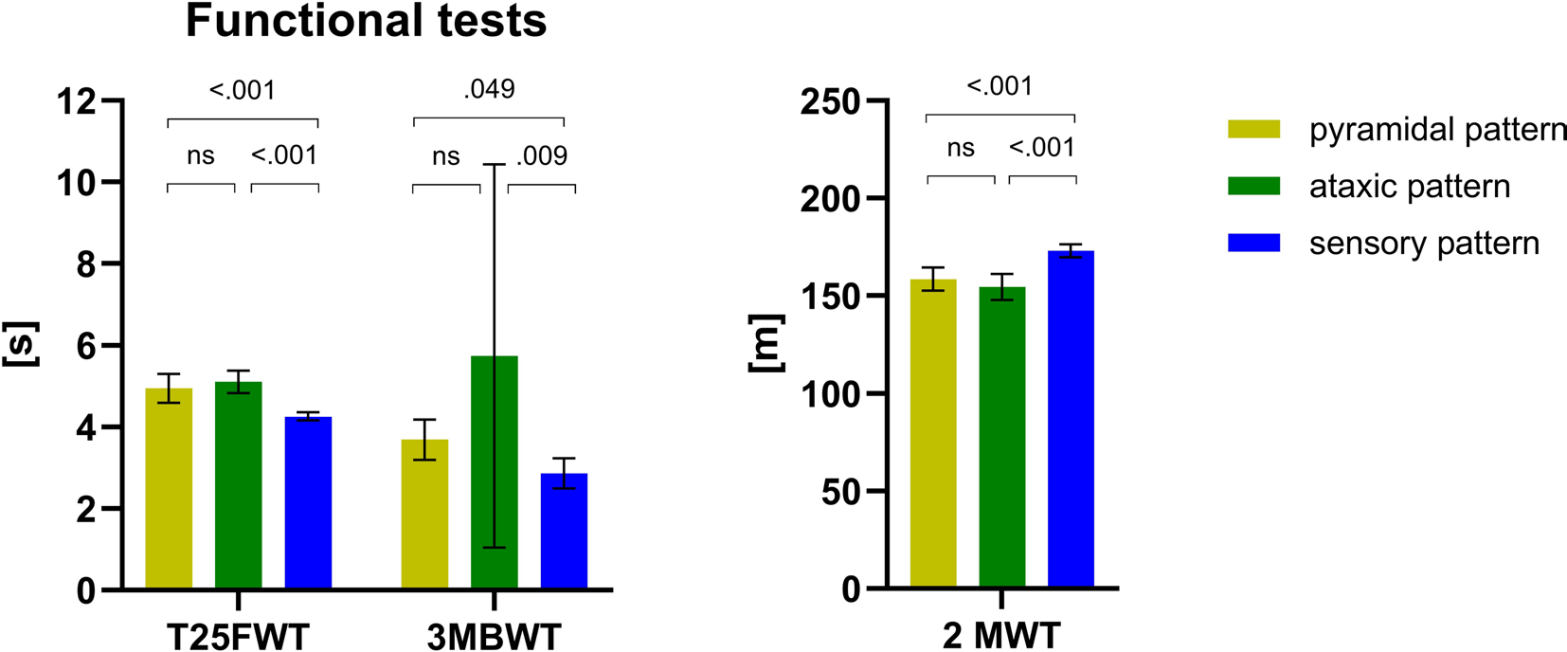
Comparison of clinical functional testing between MS gait pattern groups (pyramidal, ataxic, sensory) (N = 204). Data are presented as mean and 95% CI. For the 3MBWT, a high proportion of missing values (≈ 70–80%) in all groups and small subgroup size in the ataxic group (*n* = 4) led to wide confidence intervals, which should be interpreted with caution; p-values between the three MS patterns (Kruskal Wallis analysis; pairwise comparisons with Mann-Whitney U-Test and Bonferroni adjustment). T25FWT = Timed 25 Foot Walk Test; 3MBWT = 3 m backwards walking Test; 2 MWT = 2 min Walk Test; ns = not significant.

The three gait pattern groups exhibited similar characteristics in terms of sex and BMI distribution (all *P* > 0.05). However, patients with a sensory gait pattern tended to be younger compared to the ataxic or pyramidal gait pattern groups (*P* = 0.001) and had lower EDSS (*P* < 0.001). PwMS in the sensory group showed less impairment in function tests such as the 2-minute Walk Test (2 MWT distance: 173.01 ± 18.07 m) and 3 m backward walking (3MBWT time: 2.86 ± 0.76 s) compared to the other gait pattern groups (Fig. 2). Conversely, pwMS were more severely affected in terms of EDSS an demonstrated poorer performance in all functional tests compared to the sensory group (e.g. 2 MWT:154.41 ± 17.57 m vs. 173.01 ± 18.07 m; ataxic vs. sensory gait pattern *P* < 0.001) (Fig. 2).

The results of the functional test showed significant differences between the respective gait pattern groups (T25FWT: *P* < 0.001; 2 MWT: *P* < 0.001; 3MBWT: *P* = 0.003). Due to the high proportion of missing values in the 3MBWT (approximately 70–80%) and the small sample size in the ataxic group (*n* = 4), results from this test should be interpreted with caution, as reflected in the wide confidence intervals.

**Fig. 3:**
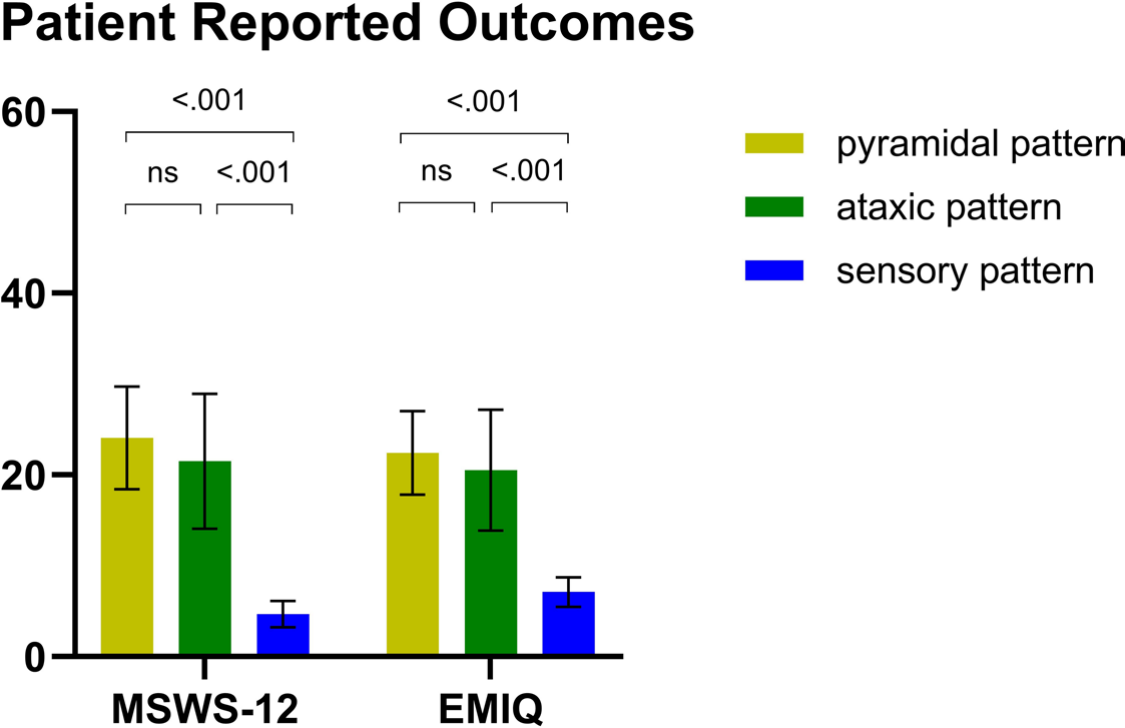
Comparison of Patient reported Outcomes between MS gait pattern groups (pyramidal, ataxic, sensory) (N = 204). Data are presented as mean and 95% CI; ^p-values between the three MS patterns (Kruskal Wallis analysis; pairwise comparisons with Mann-Whitney U-Test and Bonferroni adjustment). MSWS-12 = Multiple Sclerosis Walking Scale; EMIQ = Early Mobility Impairment Questionnaire; ns = not significant.

The patterns observed in objective clinical function tests were consistent with the results reported by the patients (Fig. 3). Specifically, pwMS with a sensory gait pattern reported less gait impairment compared to those with ataxic or pyramidal patterns (all *P* < 0.001 for MSWS-12 and EMIQ, while minor, non-statistically significant difference was observed between the latter two groups).

### Characterisation of MS gait pattern groups by specific Spatiotemporal gait parameters across different walking conditions

**Fig. 4:**
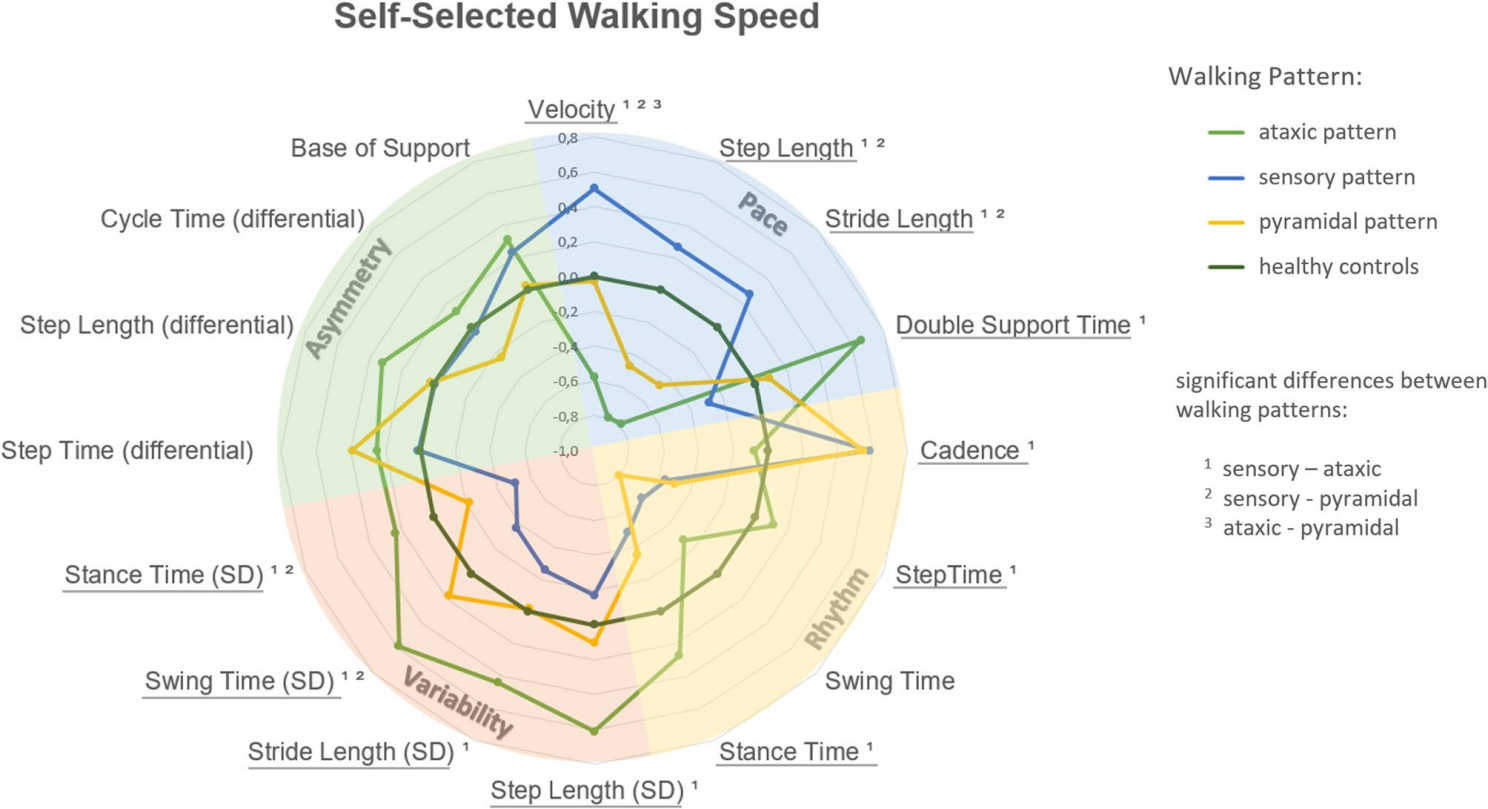
Radar plot representation of gait patterns in pwMS at self-selected speed. This plot illustrates the mean Z-scores of spatiotemporal gait variables for three gait pattern groups in people with Multiple Sclerosis (pwMS). Each pattern group is represented by a separate spider-web, while Healthy Controls are shown as a circular reference. Higher or lower Z-Scores in pwMS reveal how each group’s gait characteristics vary in relation to Healthy Controls, highlighting the severity of gait impairments. Statistical differences between the three groups were analysed using multivariable generalised linear models. Data were adjusted for age, sex, and BMI. Significant main effects are indicated by underlined gait variable names, and significant pairwise comparisons are denoted by superscripts. PwMS with an ataxic gait pattern showed markedly reduced walking speed and increased variability, whereas the sensory group demonstrated relatively preserved motor function with higher walking speed and longer step and stride lengths.

**Fig. 5:**
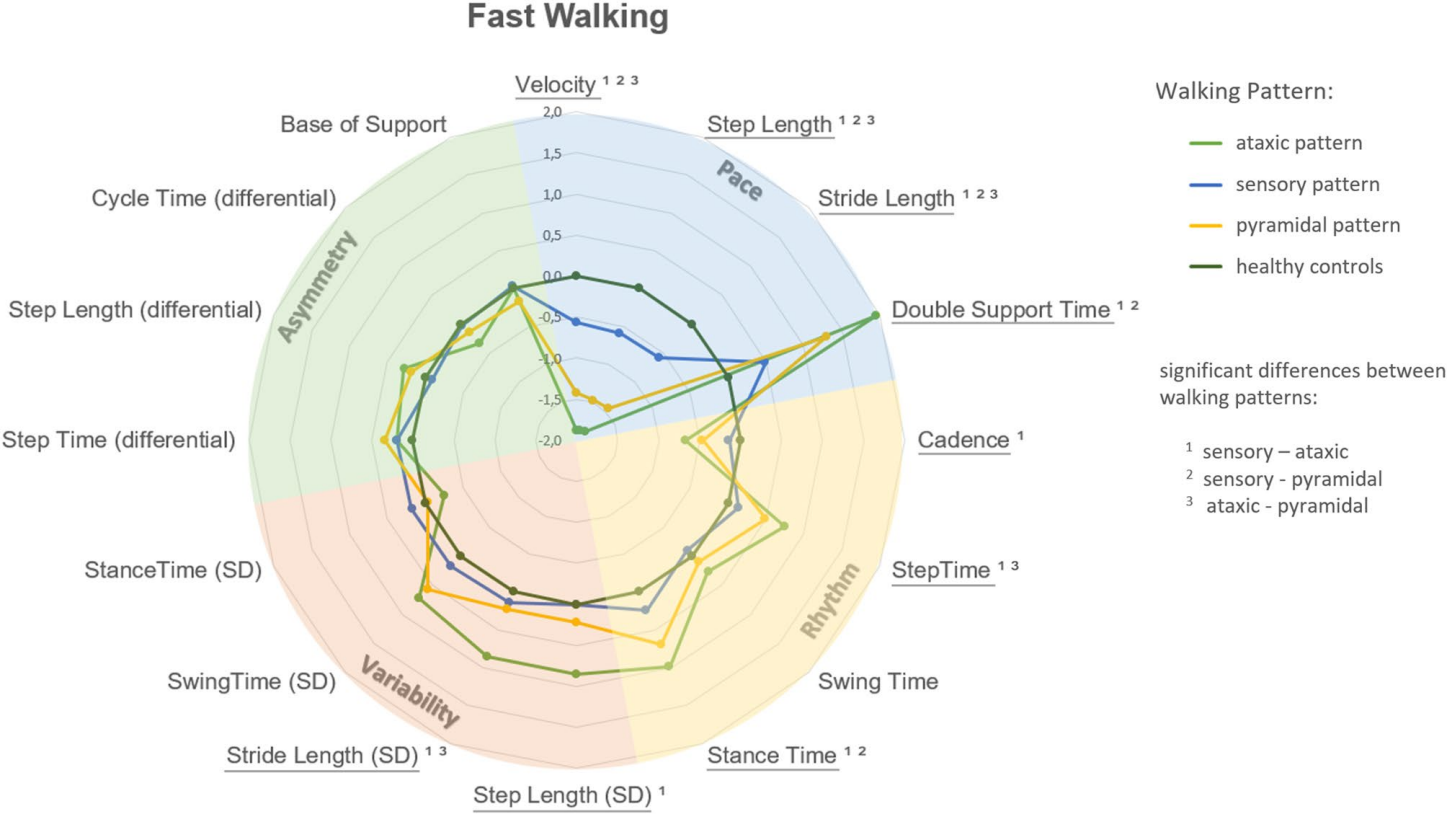
Radar plot representation of gait patterns in pwMS at fast walking speed. This plot illustrates the mean Z-scores of spatiotemporal gait variables for three gait pattern groups in people with Multiple Sclerosis (pwMS). Each pattern group is represented by a separate spider-web, while Healthy Controls are shown as a circular reference. Higher or lower Z-Scores in pwMS reveal how each group’s gait characteristics vary in relation to Healthy Controls, highlighting the severity of gait impairments. Statistical differences between the three groups were analysed using multivariable generalised linear models. Data were adjusted for age, sex, and BMI. Significant main effects are indicated by underlined gait variable names, and significant pairwise comparisons are denoted by superscripts. Ataxic gait patterns were characterised by pronounced impairments in the pace domain compared to pyramidal patterns, while sensory patterns showed relatively preserved motor function across most domains.

**Fig. 6:**
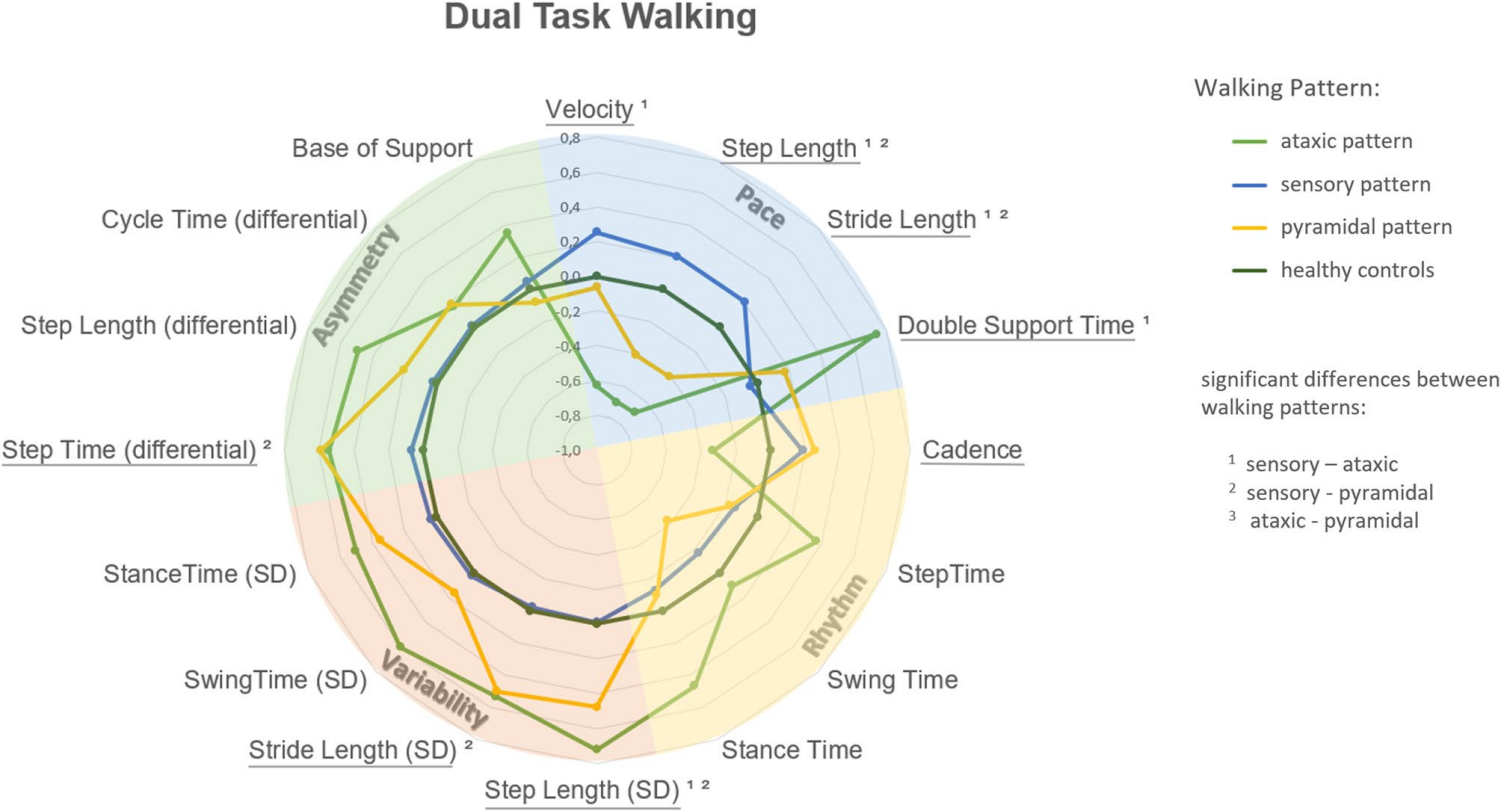
Radar plot representation of gait patterns in pwMS at dual task walking. This plot illustrates the mean Z-scores of spatiotemporal gait variables for three gait pattern groups in people with Multiple Sclerosis (pwMS). Each pattern group is represented by a separate spider-web, while Healthy Controls are shown as a circular reference. Higher or lower Z-Scores in pwMS reveal how each group’s gait characteristics vary in relation to Healthy Controls, highlighting the severity of gait impairments. Statistical differences between the three groups were analysed using multivariable generalised linear models. Data were adjusted for age, sex, and BMI. Significant main effects are indicated by underlined gait variable names, and significant pairwise comparisons are denoted by superscripts. Ataxic gait patterns demonstrated the greatest impairments with reduced step and stride length and increased variability, while sensory patterns performed significantly better than pyramidal patterns, particularly in spatial parameters.

Across all walking conditions, the three gait pattern groups (sensory, pyramidal, ataxic) showed distinct profiles in spatiotemporal parameters (Figs. 4, 5 and 6). Notably, the ataxic group consistently exhibited the greatest impairments - particularly in gait variability and asymmetry - while the sensory group showed the most preserved function. Detailed descriptive statistics for all gait parameters are provided in Supplementary Table A1.

#### Self-selected walking speed

The domains pace and variability showed significant differences between the different gait patterns of pwMS when walking at self-selected walking speed. A significant main effect was observed in pace across all gait patterns (*P* < 0.001), with post-hoc pairwise comparisons revealing significant differences between sensory-ataxic (*P* < 0.001), sensory-pyramidal (*P* = 0.007), and ataxic-pyramidal (*P* = 0.035) gait patterns. No significant differences were observed in the asymmetry domain between the gait patterns.

PwMS with an ataxic gait pattern have a significantly reduced walking speed with reduced step and stride length in comparison to the sensory and pyramidal pattern groups. Furthermore, the ataxic MS group differs from the sensory pwMS in particular in all variability parameters (Fig. 4 and Table A2 in the Supplementary material), all pace parameters and almost all rhythm parameter (except swing time).

The sensory gait pattern is characterised by a significantly increased walking speed, in combination with an increased step and stride length (sensory-ataxic: *P* < 0.001; sensory-pyramidal: *P* = 0.001) in the pace domain compared to the ataxic and pyramidal gait pattern. In the rhythm domain, this pattern shows significantly increased cadence compared to the ataxic MS gait pattern group (*P* = 0.018). In contrast, both the pyramidal and ataxic gait pattern groups show significantly reduced walking speed, as well as reduced step and stride length, compared to the sensory group.

#### Fast walking speed

Significant differences were found in the pace domain among all three gait patterns during fast walking (*P* < 0.001; Fig. 5 and Table A2 in the Supplementary material).

Post-hoc comparisons revealed that the ataxic gait pattern group exhibited significant limitations in the pace domain parameters compared to patients with the pyramidal gait pattern (with a reduced velocity: *P* = 0.040; step length: *P* = 0.029 and stride length: *P* = 0.032). Furthermore, variability measures showed significantly higher stride length variability in the ataxic compared to the pyramidal pattern (*P* = 0.026).

The sensory gait pattern group had significantly better results in the rhythm domain (with the variables cadence, step time and stance time) and in the variability domain (with the variables step length and stride length) compared to the ataxic gait pattern.

Compared to the MS group with a sensory gait pattern the pyramidal gait pattern group exhibited significantly reduced walking speed (*P* = 0.002), shorter step and stride lengths (*P* = 0.006), and a longer double support time (*P* = 0.028) in the pace domain. In contrast to walking at self-selected walking speed, fast walking revealed more significant differences between ataxic and pyramidal gait patterns, as illustrated in Figs. 4 and 5.

#### Dual task walking

During dual-task walking, significant main effects were found in the pace and variability domains during dual-task walking (*P* < 0.001; Fig. 6 and Table A2 in the Supplementary material). Post-hoc comparisons showed that the ataxic gait pattern group exhibited the most pronounced impairments, with significantly reduced step and stride lengths and increased gait variability compared to the sensory group (*P* < 0.001).

Furthermore the MS group with a sensory gait pattern demonstrated significantly better spatial performance than the pyramidal group, particularly in step and stride length (*P* = 0.004), as well as lower stride length variability (*P* = 0.021).

In addition the pwMS with a pyramidal gait pattern showed greater stride time asymmetry compared to the sensory pattern (*P* = 0.016). No significant differences were observed between the ataxic and pyramidal gait patterns under dual-task conditions.

## Discussion

This study provides a comprehensive multidimensional gait analysis to characterise three distinct clinical-based gait patterns (sensory, pyramidal, and ataxic) in pwMS. PwMS were classified based on a structured assessment of EDSS functional system subscores, suring that only lower extremity impairments contributed to group assignment. Sensory gait patterns were the most prevalent (57.8%), followed by pyramidal (27.9%) and ataxic patterns (14.2%). Our findings provide valuable insights into motor deficits associated with MS-related gait impairments, with the potential to enhance diagnostic precision and guide targeted therapeutic strategies. Gait pattern differences were evident across all walking conditions, particularly in variability, pace, rhythm, and symmetry domains.

With self-selected walking speed, pwMS with ataxic gait patterns showed significantly reduced walking speed and increased variability, indicating impaired motor control. In contrast, sensory gait patterns demonstrated relatively preserved motor function, with higher walking speed and longer step and stride lengths, suggesting intact motor pathways but impaired sensory feedback. Pyramidal gait patterns displayed moderate impairments, resembling ataxic patterns in terms of tempo, asymmetry, and rhythm, but with less pronounced variability. Spatiotemporal analysis revealed the most pronounced differences in walking speed, step length, and stride length between sensory versus ataxic gait patterns, as well as between sensory versus pyramidal gait patterns. PwMS with ataxic gait patterns exhibited the greatest impairments across all three gait conditions, characterised by significantly reduced walking speed, increased variability, and altered step and stride lengths compared to sensory or pyramidal patterns. Sensory gait patterns showed relatively preserved motor function, while pyramidal patterns demonstrated moderate impairments, particularly under fast-walking conditions. Walking at self-selected speed showed the clearest differentiation between sensory versus ataxic patterns, as well as between sensory versus pyramidal patterns In contrast, fast walking revealed a greater number of significant differences between ataxic and pyramidal patterns. This suggests that increasing walking speed may be useful for identifying specific motor deficits between gait patterns in MS, particularly in distinguishing between ataxic and pyramidal patterns. Additional studies could explore condition-condition comparisons to determine the optimal methodology for identifying motor deficits across varying gait conditions.

Our study shows that the ataxic gait pattern does not differ from the pyramidal gait pattern in any of the clinical function tests (25FWT, 2MWT) and/or patient-reported outcomes (EMIQ, MSWS-12). A detailed examination of variables in different gait domains—such as variability, pace, rhythm, and asymmetry— is required to distinguish these patterns.

These findings align with prior research that identified significant differences in spatio-temporal parameters between pwMS and HC, suggesting reduced walking speed and cadence, shorter stride lengths, and increased base of support and gait variability as hallmarks of MS-related gait impairments^38–43^. Furthermore, our results corroborate Kalron and Givon’s observations of relatively intact gait patterns in sensory impairments and greater asymmetry and slowed gait, shorter stride and poor stance stability in pyramidal dysfunctions^7^. Our results show significant differences in stance time between the sensory vs. pyramidal patterns during fast walking and in step length between the ataxic vs. pyramidal patterns across all three gait conditions and are therefore consistent with the results of the mentioned study. Furthermore, our analyses of functional tests and PROs indicate that pwMS with a sensory gait pattern perceive relatively low walking impairment. While our primary focus was on differentiating gait patterns within the MS cohort rather than direct comparisons with healthy controls (HC), we acknowledge that certain gait parameters in the sensory pattern group appeared comparable or even slightly better than in HC. This observation is not entirely unexpected, as previous studies have reported similar findings, particularly in MS patients with lower disability levels and preserved mobility. For instance we observed that pwMS with minimal disease duration and higher walking speeds exhibited gait characteristics similar to or slightly differing from HC, particularly in cadence and stride length^44^. Such effects may be attributed to compensatory mechanisms or adaptations in early disease stages. However, given that our study used HC data primarily for Z-score normalization rather than direct statistical comparisons, these observations should be interpreted cautiously.

Interestingly, our results differ from previous reports, which suggested more pronounced impairments in pyramidal than cerebellar dysfunctions^11,15^. However, the significant increased variability deficits in the ataxic pattern group aligns with studies showing impairment of bilateral gait coordination^45–47^. PwMS show impairments in dual-task performance, as indicated by a significant slowing of walking speed and changes in gait parameters such as cadence, swing time and double support time under dual-task conditions^48–50^. Our results indicate this statement - although the ataxic pattern group shows the most changes compared to the sensory pattern.

A key methodological consideration in gait analysis is the strong dependence of spatiotemporal parameters on walking speed. In this study, self-selected and fast walking conditions were used to reflect real-life variability in gait performance. However, future studies should investigate whether adjusting for walking speed or applying speed-normalised gait parameters can help to disentangle speed-dependent effects from underlying motor impairments. Such approaches have been applied in previous research to minimize confounding effects of velocity differences, allowing a clearer interpretation of gait impairments beyond mere speed reductions^39^.

Moreover, differences in gait parameters across MS subgroups may partly reflect overall disability level rather than isolated functional system impairments. Preiningerova et al. demonstrated that specific gait characteristics, such as step length and double support time, change systematically with increasing EDSS levels^39^. While this correlation is well-established, our approach accounted for this by basing classification on the three most clinically relevant functional systems (pyramidal, cerebellar, sensory), incorporating their respective lower extremity subscores in a multivariable manner. This refined EDSS-based approach may better reflect functional gait impairment patterns than prior studies that used more general EDSS categories.

Although the EDSS is known to be limited by subjectivity and reduced sensitivity to subtle gait abnormalities, its clinical relevance and widespread application make it a valuable tool in MS-related research^51^. Our method aimed to counterbalance its limitations by incorporating detailed subscores and using them not in isolation, but in a combined classification model. Future studies may enhance this further by integrating objective biomarkers such as neuroimaging or EMG-based motor profiles.

Our study focused on pwMS with mild to moderate disability (EDSS ≤ 5.5, median EDSS: 2.0), ensuring that all participants were still ambulatory without assistive devices. This is important, as gait impairment manifests differently across disability stages. PwMS with EDSS ≥ 6.0 typically require walking aids, limiting their ability to complete standard gait assessments. While our findings provide insights into ambulatory pwMS, further research is needed to examine more severely affected patients, potentially using alternative assessment tools such as wearable sensors or static balance assessments.

Another limitation is the absence of a detailed cognitive profile for the participants. Cognitive dysfunction, particularly impairments in executive function and attentional resource allocation, has been shown to significantly influence gait variability. Notably, gait variability is strongly associated with cognitive function not only in pwMS but also in other neurological disorders, including Parkinson’s disease and dementia or Amyotrophic lateral sclerosis, where increased gait variability has been linked to executive dysfunction^16,17^. Although dual-task walking in our study indirectly reflects cognitive–motor interference, explicit cognitive assessments (e.g., neuropsychological tests) would have strengthened the interpretation of our findings and helped disentangle motor from cognitive contributions to gait deficits. Future studies should systematically integrate standardized cognitive assessments and multimodal measures to better capture these interactions.

The primary aim of this study was the phenotypic classification of gait patterns in pwMS, guided by detailed EDSS subscores reflecting the current functional status. This approach provides a robust basis for defining clinically meaningful gait phenotypes. While variables such as disease duration, relapse history, and DMT exposure were not systematically controlled for in the statistical analyses, information on disease duration and DMT use has been included in the descriptive characterization of the cohorts (Table 2). These additional data provide a more complete overview of the study population, while ensuring that the focus of our analyses remained on current functional manifestations rather than historical or treatment-related factors. Future studies may extend this approach by systematically integrating such variables to explore their interaction with gait phenotypes.

Another limitation is that time-of-day variations in gait impairment were not accounted for. Despite this, studies have favourably evaluated the test-retest reliability of gait characteristics across various gait domains^52^. A strength of this study is the inclusion of dual-task walking to enhance ecological validity, as it better reflects real-world cognitive-motor demands. Nevertheless, assessments were still performed under controlled laboratory conditions, which cannot fully replicate the complexity of real-world environments (e.g., uneven terrain, unpredictable obstacles). Future studies should address this limitation by integrating wearable sensor-based assessments in free-living environments to capture context-dependent gait variability.

The gait pattern in pwMS is most commonly characterised as paretic-spastic, cerebellar, or sensory, each of which has specific implications for subsequent symptomatic therapy and rehabilitation strategies. Focusing symptomatic therapy on these patterns can effectively improve the gait pattern of pwMS and should therefore be included in multidisciplinary treatment programs^53^. However, such detailed gait characterisation is only possible through specific gait analysis. This clinical relevance of gait pattern differentiation lies in its potential for individualized rehabilitation strategies. PwMS with pyramidal gait patterns may benefit from strength and endurance training, alongside spasticity management. Ataxic gait patterns often require coordination and balance training, incorporating task-oriented exercises or core stabilization techniques. Sensory gait patterns may respond well to proprioceptive and balance training to enhance gait stability^37,54^. Depending on the gait phenotype, future rehabilitation approaches might use pattern-specific spatiotemporal parameters as quantitative outcome markers to evaluate the effectiveness of targeted interventions. These tailored approaches highlight the importance of integrating biomechanical and clinical insights into rehabilitation planning.

Given the multifactorial nature of MS-related gait impairment, some overlap between gait patterns is inevitable. While our classification approach aimed to capture distinct gait phenotypes, certain pwMS may exhibit characteristics spanning multiple functional systems. For example, the ataxic group may present with mild pyramidal involvement, while the sensory group may show minor cerebellar deficits. However, our classification criteria were designed to prioritise the predominant gait impairment pattern while minimising heterogeneity within each subgroup. To further refine gait phenotyping in pwMS, future studies should explore alternative classification methods, such as data-driven clustering approaches or machine learning (ML). Automated pattern recognition technologies have already been successfully applied in other neurological disorders, such as positional vertigo, cerebellar ataxia, and progressive supranuclear palsy but have not yet been extensively used in pwMS^55,56^.

Beyond classification, understanding how gait patterns evolve over time is critical. Longitudinal studies are essential to track changes in gait characteristics and identify prognostic markers for disease progression. Prior research, such as Filli et al. (2018), demonstrated that pwMS with spastic-paretic gait patterns experience the greatest decline in walking performance, particularly in the 6MWT, with reduced knee flexion as a key contributor^15^. While these findings provide valuable insights, our study differs by linking gait patterns directly to EDSS-defined functional system scores, enabling a more direct correlation between neurological impairment and gait phenotype.

Future research should build upon these findings by integrating multimodal assessment strategies. Wearable sensor technology could enable continuous, real-world gait monitoring, while neuroimaging techniques may provide deeper insights into structural and functional brain changes associated with gait impairment. Additionally, examining compensatory movement strategies and gait variability in response to disease progression could help identify early markers of functional decline. By incorporating these approaches, future studies could enhance both the characterisation of MS-related gait patterns and the development of targeted, pattern-specific interventions to preserve or improve mobility in pwMS.

## Conclusion

The differentiated characterisation of gait patterns in pwMS demonstrates that specific impairments in distinct functional systems lead to clinically relevant gait alterations, which are often difficult to detect without detailed gait analyses. These findings highlight the need for integrating instrumented gait assessment into routine clinical practice to improve the precision of classification and enable the development of phenotype-specific rehabilitation strategies. Beyond cross-sectional insights, future research should establish longitudinal protocols to monitor gait changes over time, identify early markers of disease progression, and assess the effectiveness of targeted interventions. Combining sensor-based gait analysis with cognitive assessments, disease-related variables, and patient-reported outcomes will further enhance the ecological validity and clinical applicability of gait phenotyping. Ultimately, embedding these approaches into an MS monitoring framework could support personalised treatment planning and adaptive rehabilitation strategies, moving towards a data-driven and patient-centred model of care^57^.

## Supplementary Information

Below is the link to the electronic supplementary material.

Supplementary Material 1

## Data Availability

The datasets used and analysed during the current study available from the corresponding author on reasonable request.

## References

[1] Weinshenker, B. G. et al. The natural history of multiple sclerosis: A geographically based study. *Brain* **112**, 133–146 (1989). 2917275 10.1093/brain/112.1.133

[2] Disanto, G. et al. Heterogeneity in multiple sclerosis: scratching the surface of a complex disease autoimmune diseases. *Autoimmune Dis.* 932351. 10.4061/2011/932351 (2011). 10.4061/2011/932351 PMC3005811 21197462

[3] LaRocca, N. G. Impact of walking impairment in multiple sclerosis. *Patient Patient-Centered Outcomes Res.* **4**, 189–201 (2011). 10.2165/11591150-000000000-00000 21766914

[4] Sosnoff, J. J., Gappmaier, E., Frame, A. & Motl, R. W. Influence of spasticity on mobility and balance in persons with multiple sclerosis. *J. Neurol. Phys. Ther.* **35**, 129–132 (2011). 21934374 10.1097/NPT.0b013e31822a8c40

[5] Kalron, A. & Frid, L. Journal of the neurological sciences the butterflydiagram: A gait marker for neurological and cerebellar impairment in people with multiple sclerosis. *J. Neurol. Sci.* **358**, 92–100 (2015). 26318202 10.1016/j.jns.2015.08.028

[6] Krbot Skorić, M., Crnošija, L., Gabelić, T., Adamec, I. & Habek, M. Relationship between sensory dysfunction and walking speed in patients with clinically isolated syndrome. *J. Clin. Neurophysiol.* **35**, 65–70 (2018). 29135702 10.1097/WNP.0000000000000431

[7] Kalron, A. & Givon, U. Gait characteristics according to pyramidal, sensory and cerebellar EDSS subcategories in people with multiple sclerosis. *J. Neurol.* **263**, 1796–1801 (2016). 27314963 10.1007/s00415-016-8200-6

[8] Meyer-Moock, S., Feng, Y. S., Maeurer, M., Dippel, F. W. & Kohlmann, T. Systematic literature review and validity evaluation of the expanded disability status scale (EDSS) and the multiple sclerosis functional composite (MSFC) in patients with multiple sclerosis. *BMC Neurol.* **14**, 58 (2014). 24666846 10.1186/1471-2377-14-58 PMC3986942

[9] Angelini, L. et al. A multifactorial model of multiple sclerosis gait and its changes across different disability levels. *IEEE Trans. Biomed. Eng.* **68**, 3196–3204 (2021). 33625975 10.1109/TBME.2021.3061998

[10] Monaghan, A. S., Huisinga, J. M. & Peterson, D. S. The application of principal component analysis to characterize gait and its association with falls in multiple sclerosis. *Sci. Rep.* **11**, 1–10 (2021). 34140612 10.1038/s41598-021-92353-2 PMC8211858

[11] Coca-Tapia, M., Cuesta-Gómez, A. & Molina-Rueda, F. Carratalá-Tejada, M. Gait pattern in people with multiple sclerosis: A systematic review. *Diagonstics* **11**, 584 (2021). 10.3390/diagnostics11040584 PMC8064080 33805095

[12] Chee, J. N. et al. Influence of multiple sclerosis on Spatiotemporal gait parameters: A systematic review and Meta-Regression. *Arch. Phys. Med. Rehabil*. 10.1016/j.apmr.2020.12.013 (2021). 33460576 10.1016/j.apmr.2020.12.013

[13] Sato, S. D. et al. Spatiotemporal gait changes in people with multiple sclerosis with different disease progression subtypes. *Clin. Biomech.* **100**, 105818 (2022). 10.1016/j.clinbiomech.2022.105818 36435079

[14] Dujmovic, I. et al. Gait pattern in patients with different multiple sclerosis phenotypes. *Mult Scler. Relat. Disord*. **13**, 13–20 (2017). 28427694 10.1016/j.msard.2017.01.012

[15] Filli, L. et al. Profiling walking dysfunction in multiple sclerosis: characterisation, classification and progression over time. *Sci. Rep.* **8**, 1–13 (2018). 29563533 10.1038/s41598-018-22676-0 PMC5862880

[16] Dubbioso, R. et al. Cognitive impairment is associated with gait variability and fall risk in amyotrophic lateral sclerosis. *Eur. J. Neurol.* **30**, 3056–3067 (2023). 37335396 10.1111/ene.15936

[17] Spisto, M. et al. Identifying mild behavioral and neurocognitive impairment in amyotrophic lateral sclerosis (MBNI-ALS) provides key prognostic insights. *Eur. J. Neurol.* **32**, 1–12 (2025). 10.1111/ene.70171 PMC12045931 40312886

[18] Thompson, A. J. et al. Diagnosis of multiple sclerosis: 2017 revisions of the McDonald criteria. *Lancet Neurol. V*. **17**, 162–173 (2018). 10.1016/S1474-4422(17)30470-2 29275977

[19] Trentzsch, K. et al. The Dresden protocol for multidimensional walking assessment (DMWA) in clinical practice. *Front. Neurosci.* **14**, (2020). 10.3389/fnins.2020.582046 PMC7649388 33192268

[20] Kurtzke, J. F. & Hutchinson, M. Rating neurologic impairment in multiple sclerosis: an expanded disability status scale (EDSS). *Neurology* **33**, 1444–1452 (1983). 6685237 10.1212/wnl.33.11.1444

[21] Rowling, M., Crockford, G. P., Clairmont, C. & Hassel, J. F. N. *GAITRite-Handbuch Version 4.7*. (2012).

[22] Learmonth, Y. C., Ensari, I. & Motl, R. W. Cognitive motor interference in multiple sclerosis: insights from a systematic quantitative review. *Arch. Phys. Med. Rehabil*. **98**, 1229–1240 (2017). 27543046 10.1016/j.apmr.2016.07.018

[23] Leone, C., Patti, F. & Feys, P. Measuring the cost of cognitive-motor dual tasking during walking in multiple sclerosis. *Mult Scler. J.* **21**, 123–131 (2015). 10.1177/1352458514547408 25178543

[24] Wajda, D. A. & Sosnoff, J. J. Cognitive-motor interference in multiple sclerosis: a systematic review of evidence, correlates, and consequences. *Biomed. Res. Int.* **2015** 720856 (2015). 10.1155/2015/720856 PMC4369906 25839039

[25] Hollman, J. H., McDade, E. M. & Petersen, R. C. Normative Spatiotemporal gait parameters in older adults. *Gait Posture*. **34**, 111–118 (2011). 21531139 10.1016/j.gaitpost.2011.03.024 PMC3104090

[26] Lord, S. et al. Independent domains of gait in older adults and associated motor and nonmotor attributes: validation of a factor analysis approach. *Journals Gerontol. - Ser. Biol. Sci. Med. Sci.* **68**, 820–827 (2013). 10.1093/gerona/gls255 23250001

[27] Verghese, J., Wang, C., Lipton, R. B., Holtzer, R. & Xue, X. Quantitative gait dysfunction and risk of cognitive decline and dementia. *J. Neurol. Neurosurg. Psychiatry*. **78**, 929–935 (2007). 17237140 10.1136/jnnp.2006.106914 PMC1995159

[28] Verghese, J. et al. Gait dysfunction in mild cognitive impairment syndromes. *J. Am. Geriatr. Soc.* **56**, 1244–1251 (2008). 18482293 10.1111/j.1532-5415.2008.01758.x PMC2574944

[29] Cutter, G. R. et al. Development of a multiple sclerosis functional composite as a clinical trial outcome measure. *Brain* **122**, 871–882 (1999). 10355672 10.1093/brain/122.5.871

[30] Gijbels, D., Eijnde, B. O. & Feys, P. Comparison of the 2- and 6-minute walk test in multiple sclerosis. *Mult Scler.* **17**, 1269–1272 (2011). 21642370 10.1177/1352458511408475

[31] Scalzitti, D. A. et al. Validation of the 2-Minute walk test with the 6-Minute walk test and other functional measures in persons with multiple sclerosis. *Int. J. MS Care*. **20**, 158–163 (2018). 30150899 10.7224/1537-2073.2017-046 PMC6107337

[32] Edwards, E. M., Daugherty, A. M., Nitta, M., Atalla, M. & Fritz, N. E. Backward walking sensitively detects fallers in persons with multiple sclerosis. *Mult Scler. Relat. Disord*. **45**, 102390 (2020). 32707529 10.1016/j.msard.2020.102390

[33] Stölzer-Hutsch, H., Schriefer, D., Trentzsch, K. & Ziemssen, T. Backward and forward walking and its association with falls and fear of falling in people with multiple sclerosis. *Gait Posture*. **106**, S197 (2023).

[34] Mokkink, L. B., Galindo-Garre, F. & Uitdehaag, B. M. Evaluation of the multiple sclerosis walking Scale-12 (MSWS-12) in a Dutch sample: application of item response theory. *Mult Scler. J.* **22**, 1867–1873 (2016). 10.1177/1352458516630821 26873891

[35] Ziemssen, T. et al. Development of the multiple sclerosis (MS) early mobility impairment questionnaire (EMIQ). *J. Neurol.* **263**, 1969–1983 (2016). 27393117 10.1007/s00415-016-8210-4

[36] Voisard, C. et al. Innovative multidimensional gait evaluation using IMU in multiple sclerosis: introducing the semiogram. *Front. Neurol.* **14**, (2023). 10.3389/fneur.2023.1237162 PMC10540441 37780706

[37] Hemmer, B. & Gehring, K. Diagnose und Therapie der Multiplen Sklerose, Neuromyelitis-optica- Spektrum-Erkrankungen und MOG-IgG-assoziierten Erkrankungen: S2k-Leitlinie. *Deutsche Gesellschaft für Neurologie* www.dgn.org/leitlinien (2024).

[38] Comber, L., Galvin, R. & Coote, S. Gait deficits in people with multiple sclerosis: A systematic review and meta-analysis. *Gait Posture*. **51**, 25–35 (2017). 27693958 10.1016/j.gaitpost.2016.09.026

[39] Preiningerova, J. L. et al. Spatial and Temporal characteristics of gait as outcome measures in multiple sclerosis (EDSS 0 to 6.5). *J. Neuroeng. Rehabil.* **12**, (2015). 10.1186/s12984-015-0001-0 PMC4334845 25890382

[40] Sacco, R., Bussman, R., Oesch, P., Kesselring, J. & Beer, S. Assessment of gait parameters and fatigue in MS patients during inpatient rehabilitation: a pilot trial. *J. Neurol.* **258**, 889–894 (2011). 21076978 10.1007/s00415-010-5821-z

[41] Crenshaw, S. J., Royer, T. D., Richards, J. G. & Hudson, D. J. Gait variability in people with multiple sclerosis. *Mult Scler. J.* **12**, 613–619 (2006). 10.1177/1352458505070609 17086908

[42] Huisinga, J. M., Mancini, M., George, S., Horak, F. B. & R. J. & Accelerometry reveals differences in gait variability between patients with multiple sclerosis and healthy controls. *Ann. Biomed. Eng.* **41**, 1670–1679 (2013). 23161166 10.1007/s10439-012-0697-y PMC3987786

[43] Plotnik, M., Wagner, J. M., Adusumilli, G., Gottlieb, A. & Naismith, R. T. Gait asymmetry, and bilateral coordination of gait during a six-minute walk test in persons with multiple sclerosis. *Sci. Rep.* **10**, (2020). 10.1038/s41598-020-68263-0 PMC7382471 32709914

[44] Inojosa, H. et al. Characterization of early gait alterations in multiple sclerosis using gaitrite analysis in a large cohort. *Mult. Scler. J.* **29** 445 (2023).

[45] Correale, L. et al. Bilateral coordination of gait at self-selected and fast speed in patients with multiple sclerosis: a case-control study. *Mult. Scler. Relat. Disord.* **65**, (2022). 10.1016/j.msard.2022.104027 35810720

[46] Richmond, S. B., Swanson, C. W., Peterson, D. S. & Fling, B. W. A Temporal analysis of bilateral gait coordination in people with multiple sclerosis. *Mult Scler. Relat. Disord*. **45**, 102445 (2020). 32791490 10.1016/j.msard.2020.102445

[47] Kalron, A. & Frid, L. The butterfly diagram: A gait marker for neurological and cerebellar impairment in people with multiple sclerosis. *J. Neurol. Sci.* **358**, 92–100 (2015). 26318202 10.1016/j.jns.2015.08.028

[48] Hamilton, F. et al. Walking and talking: an investigation of cognitive-motor dual tasking in multiple sclerosis. *Mult Scler.* **15**, 1215–1227 (2009). 19667011 10.1177/1352458509106712

[49] Allali, G., Laidet, M., Assal, F., Armand, S. & Lalive, P. H. Walking while talking in patients with multiple sclerosis: the impact of specific cognitive loads. *Neurophysiol. Clin. Neurophysiol.* **44**, 87–93 (2014). 10.1016/j.neucli.2013.10.136 24502909

[50] Kalron, A., Dvir, Z. & Achiron, A. Walking while talking-Difficulties incurred during the initial stages of multiple sclerosis disease process. *Gait Posture*. **32**, 332–335 (2010). 20594850 10.1016/j.gaitpost.2010.06.002

[51] Inojosa, H., Schriefer, D. & Ziemssen, T. Clinical outcome measures in multiple sclerosis: A review. *Autoimmun. Rev.* **19**, 102512 (2020). 32173519 10.1016/j.autrev.2020.102512

[52] Santinelli, F. B. et al. Between-Day reliability of the gait characteristics and their changes during the 6-Minute walking test in people with multiple sclerosis. *Neurorehabil Neural Repair.*10.1177/15459683231222412 (2024). 38229519 10.1177/15459683231222412

[53] Soler, B., Ramari, C., Valet, M., Dalgas, U. & Feys, P. Clinical assessment, management, and rehabilitation of walking impairment in MS: an expert review. *Expert Rev. Neurother.* **20**, 875–886 (2020). 32729742 10.1080/14737175.2020.1801425

[54] Tholen, R. et al. Bewegungstherapie Zur verbesserung der mobilität von patienten Mit multipler sklerose. *Neurol. Und Rehabil*. **25**, 3–40 (2020).

[55] Pradhan, C. et al. Automated classification of neurological disorders of gait using spatio-temporal gait parameters. *J. Electromyogr. Kinesiol.* **25**, 413–422 (2015). 25725811 10.1016/j.jelekin.2015.01.004

[56] Joyseeree, R., Abou Sabha, R. & Mueller, H. Applying machine learning to gait analysis data for disease identification. *Stud. Health Technol. Inf.* **210**, 850–854 (2015). 25991275

[57] Voigt, I., Inojosa, H., Wenk, J., Akgün, K. & Ziemssen, T. Building a monitoring matrix for the management of multiple sclerosis. *Autoimmun. Rev.* **22**, 103358 (2023). 37178996 10.1016/j.autrev.2023.103358

