# Supplementary material for: Phenotyping and characterising gait profiles of people with multiple sclerosis
Source: Sci Rep. 2025 Sep 17;15:32582. doi: 10.1038/s41598-025-19559-6 (PMC12443981; doi:10.1038/s41598-025-19559-6)
Supplement: Supplementary file 1 — Supplementary Material 1 [file 41598_2025_19559_MOESM1_ESM.pdf]

Table A1 *descriptive characteristics of the raw (unstandardised) values*

	**Self-Selected Walking**		
	**pyramidal pattern (N=57)**	**ataxic pattern (N= 29)**	**sensory pattern (N=118)**
**Pace**			
Velocity (cm/s)	127.25 ± 22.02 |129.30	117.70 ± 19.70 |117.90	136.50 ± 17.16 |135.65
Step Length (cm)	64.14 ± 7.57 |65.14	62.09 ± 6.66 |61.40	68.86 ± 6.30 |68.59
Double Support (s)	0.24 ± 0.07 |0.23	0.26 ± 0.08 |0.24	0.22 ± 0.04 |0.22
**Rhythm**			
Cadence (step/min)	118.54 ± 12.05 |119.90	113.20 ± 11.66 |113.80	118.86 ± 9.62 |118.45
Step Time (s)	0.51 ± 0.06 |0.50	0.54 ± 0.08 |0.53	0.51 ± 0.04 |0.51
Stance Time (s)	0.63 ± 0.09 |0.61	0.67 ± 0.11 |0.65	0.62 ± 0.06 |0.61
**Variability (SD)**			
Stride length variability	3.97 ± 2.54 |3.39	4.73 ± 2.39 |4.21	3.44 ± 1.18 |3.22
Swing time variability	0.01 ± 0.01 |0.13	0.02 ± 0.01 |0.01	0.01 ± 0.00 |0.01
**Asymmetry**			
**(difference)**	0.02 ± 0.04 |0.01	0.02 ± 0.02 |0.01	0.01 ± 0.01 |0.01
Step time asymmetry	0.01 ± 0.02 |0.00	0.01 ± 0.01 |0.01	0.01 ± 0.00 |0.01
Cycle time asymmetry	1.78 ± 1.68 |1.51	2.45 ± 2.70 |1.29	1.52 ± 1.27 |1.33
Step Length asymmetry	9.55 ± 2.58 |9.26	10.25 ± 3.11 |9.82	10.06 ± 2.69 |9.62
Base of support (cm)			

	**Fast Walking**		
	**pyramidal pattern (N=50)**	**ataxic pattern (N= 22)**	**sensory pattern (N=109)**
**Pace**			
Velocity (cm/s)	163.07 ± 28.67 |166.20	152.59 ± 23.35 |151.20	182.46 ± 22.42 |181.70
Step Length (cm)	72.71 ± 8.99 |73.22	69.68 ± 6.44 |69.34	79.44 ± 8.34 |78.77
Double Support (s)	0.17 ± 0.06 |0.17	0.19 ± 0.06 |0.18	0.15 ± 0.03 |0.15
**Rhythm**			
Cadence (step/min)	134.06 ± 14.82 |134.90	131.35 ± 15.66 |133.35	138.03 ± 11.80 |136.70
Step Time (s)	0.45 ± 0.05 |0.44	0.46 ± 0.06 |0.45	0.44 ± 0.04 |0.44
Stance Time (s)	0.54 ± 0.08 |0.53	0.56 ± 0.08 |0.54	0.51 ± 0.05 |0.52
**Variability (SD)**			
Stride length variability	3.68 ± 3.62 |3.05	5.37 ± 5.25 |4.31	3.22 ± 1.30 |2.86
Swing time variability	0.01 ± 0.02 |0.01	0.02 ± 0.05 |0.01	0.01 ± 0.00 |0.01
**Asymmetry**			
**(difference)**	0.02 ± 0.02 |0.01	0.02 ± 0.03 |0.01	0.01 ± 0.01 |0.01
Step time asymmetry	0.01 ± 0.02 |0.01	0.01 ± 0.00 |0.01	0.01 ± 0.01 |0.01
Cycle time asymmetry	2.65 ± 2.10 |2.22	3.43 ± 2.93 |2.68	2.09 ± 1.70 |1.65
Step Length asymmetry	9.79 ± 2.63 |9.53	10.26 ± 3.84 |9.68	10.35 ± 2.97 |10.05
Base of support (cm)			

	**Dual Task Walking**		
	**pyramidal pattern (N=57)**	**ataxic pattern (N= 29)**	**sensory pattern (N=118)**
**Pace**			
Velocity (cm/s)	108.89 ± 28.77 |110.10	97.25 ± 20.82 |99.50	115.38 ± 22.68 |117.45
Step Length (cm)	59.90 ± 8.36 |60.95	57.79 ± 5.96 |57.84	64.33 ± 7.03 |63.87
Double Support (s)	0.29 ± 0.10 |0.27	0.33 ± 0.11 |0.30	0.28 ± 0.08 |0.26
**Rhythm**			
Cadence (step/min)	108.02 ± 17.91 |110.50	100.57 ± 16.82 |101.20	107.17 ± 15.34 |109.45
Step Time (s)	0.58 ± 0.13 |0.54	0.62 ± 0.13 |0.59	0.57 ± 0.10 |0.55
Stance Time (s)	0.72 ± 0.17 |0.68	0.78 ± 0.17 |0.72	0.71 ± 0.15 |0.68
**Variability (SD)**			
Stride length	6.08 ± 2.18 |5.56	6.59 ± 3.51 |6.10	5.16 ± 2.77 |4.50
variability	0.03 ± 0.02 |0.21	0.05 ± 0.06 |0.03	0.03 ± 0.09 |0.02
Swing time variability			
**Asymmetry**			
**(difference)**	0.05 ± 0.18 |0.02	0.04 ± 0.08 |0.02	0.02 ± 0.03 |0.01
Step time asymmetry	0.03 ± 0.18 |0.01	0.01 ± 0.03 |0.01	0.01 ± 0.02 |0.01
Cycle time asymmetry	2.09 ± 1.59 |1.72	3.24 ± 2.59 |2.75	1.82 ± 1.44 |1.59
Step Length asymmetry	10.00 ± 2.79 |9.86	11.27 ± 4.01 |10.39	10.37 ± 2.99 |10.27
Base of support (cm)			

Scores are presented as mean ± standard deviation|median (Abbreviations: SD= standard deviation)

Table A2 *Association between Gait Variables and Gait Pattern Groups*

		pattern group	sensory- ataxic	sensory-pyramidal	ataxic-pyramidal
Self-Selected Walking					
Pace	Velocity (cm/s)	<0.001	<0.001	0.007	0.035
	Step Length (cm)	<0.001	<0.001	0.001	0.097
	Stride Length (cm)	<0.001	<0.001	0.001	0.105
	Double Support (s)	<0.001	0.002	0.059	0.115
Rhythm	Cadence (step/min)	0.022	0.018	0.288	0.156
	Step Time (s)	0.013	0.012	0.192	0.162
	Swing Time (s)	0.207			
	Stance Time (s)	0.004	0.005	0.154	0.154
Variability (SD)	Step Length variability	0.002	0.002	0.150	0.063
	Stride Length variability	0.004	0.004	0.156	0.090
	Swing Time variability	<0.001	<0.001	0.000	0.083
	Stance Time variability	<0.001	0.001	0.022	0.087
Asymmetry (difference)	Step time asymmetry	0.050			
	Step Length asymmetry	0.412			
	Cycle time asymmetry	0.254			
	Base of support (cm)	0.641			
Fast Walking					
		**pattern group**	**sensory- ataxic**	**sensory-pyramidal**	**ataxic-pyramidal**
Pace	Velocity (cm/s)	<0.001	<0.001	0.002	0.040
	Step Length (cm)	<0.001	<0.001	0.006	0.029
	Stride Length (cm)	<0.001	<0.001	0.006	0.032
	Double Support (s)	<0.001	0.000	0.028	0.060
Rhythm	Cadence (step/min)	0.032	0.077	0.096	0.495
	Step Time (s)	0.014	0.044	0.061	0.439
	Swing Time (s)	0.137			
	Stance Time (s)	0.004	0.015	0.033	0.339
Variability (SD)	Step Length variability	0.013	0.016	0.531	0.046
	Stride length variability	0.016	0.026	0.998	0.026
	Swing time variability	0.102			
	Stance Time variability	0.088			
Asymmetry (difference)	Step time asymmetry	0.501			
	Step Length asymmetry	0.111			
	Cycle time asymmetry	0.493			
	Base of support (cm)	0.853			
Dual Task Walking					
		**pattern group**	**sensory- ataxic**	**sensory-pyramidal**	**ataxic-pyramidal**
Pace	Velocity (cm/s)	0.005	0.003	0.207	0.073
	Step Length (cm)	<0.001	0.000	0.004	0.128
	Stride Length (cm)	<0.001	0.000	0.004	0.134
	Double Support (s)	0.045	0.039	0.386	0.189
Rhythm	Cadence (step/min)	0.166			
	Step Time (s)	0.258			
	Swing Time (s)	0.090			
	Stance Time (s)	0.166			
Variability (SD)	Step Length variability	0.003	0.014	0.032	0.365
	Stride length variability	0.007	0.057	0.021	0.978
	Swing time variability	0.058			
	Stance Time variability	0.209			
Asymmetry (difference)	Step time asymmetry	0.009	0.088	0.016	0.820
	Step Length asymmetry	0.070			
	Cycle time asymmetry	0.695			
	Base of support (cm)	0.223			

Multivariable generalized linear mixed models were used to analyse differences in gait parameters across the pattern groups (ataxic, sensory, and pyramidal). For each condition (self-selected walking speed, fast-walking, dual-task walking), this table presents p-values for the (fixed) main effects of pattern group variables and Bonferroni-corrected pairwise comparisons, adjusted for age, gender, and body mass index.
